# A novel assessment for Readiness Evaluation during Simulated Dismounted Operations: A reliability study

**DOI:** 10.1371/journal.pone.0226386

**Published:** 2019-12-30

**Authors:** Christopher A. Rábago, Riley C. Sheehan, Kelly A. Schmidtbauer, Michael C. Vernon, Jason M. Wilken

**Affiliations:** 1 Department of Rehabilitation Medicine, Center for the Intrepid, Brooke Army Medical Center, JBSA, Ft. Sam Houston, Texas, United States of America; 2 Extremity Trauma and Amputation Center of Excellence, JBSA, Ft. Sam Houston, Texas, United States of America; 3 Department of Rehabilitation Medicine, Uniformed Services University, Bethesda, Maryland, United States of America; 4 Henry M. Jackson Foundation for the Advancement of Military Medicine, Bethesda, Maryland, United States of America; University of Colorado Boulder, UNITED STATES

## Abstract

**Objective:**

To determine the intersession reliability of the Readiness Evaluation during Simulated Dismounted Operations (REDOp), a novel ecologically-based assessment for injured Service Members, provide minimal detectable change values, and normative reference range values. To evaluate the ability to differentiate performance limitations between able-bodied and injured individuals using the REDOp.

**Design:**

Repeated measures design and between group comparison.

**Setting:**

Outpatient rehabilitative care setting.

**Participants:**

Service Members who were able-bodied (n = 32) or sustained a traumatic lower extremity injury (n = 22).

**Interventions:**

During the REDOp, individuals walked over variable terrain as speed and incline progressively increased; they engaged targets; and carried military gear.

**Main outcome measures:**

Endurance measured using total distance traveled; walking stability measured using range of full-body angular momentum; and shooting accuracy, precision, reaction time and acquisition time.

**Results:**

Intersession reliability analyses were conducted on a sub-group of 18 able-bodied Service Members. Interclass correlation coefficient values were calculated for distance traveled (0.91), range of angular momentum about three axes (0.78–0.93), shooting accuracy (0.61), precision (0.47), reaction time (0.21), and acquisition time (0.77). Service Members with lower extremity injury demonstrated significantly less distance traveled with a median distance of 0.89 km compared to 2.73 km for the able-bodied group (p < 0.001). Service Members with lower extremity injury demonstrated significantly less stability in the frontal and sagittal planes than the able-bodied group (p < 0.001). The primary performance limiter was endurance followed by pain for both groups. There was no evidence of ceiling effects.

**Conclusions:**

The REDOp is a highly reliable, military-relevant assessment that can be used to measure performance and identify deficits across the domains of activity tolerance, gait stability, and shooting performance.

## Introduction

Mission readiness is a top priority of the U.S. Military. In military environments, multi-task activities are abundant, and functional impairments can result in loss of situational awareness and limit occupational performance, which can ultimately endanger the mission, and the lives of Service Members (SMs) and civilians. Thus, there is critical importance in identifying impairments that may restrict occupational performance and readiness.

It is difficult to objectively determine when injured SMs have successfully completed rehabilitation and are ready to return to occupational duties using current measures. Standard clinical assessment measures often lack face validity with SMs and their leaders, due to the difficulty relating performance on these tests to future performance on military-specific occupational tasks.[1] For example, the 6-minute walk test does not necessarily provide information about a SMs ability to march for several miles. Further, much of what is used lacks validity and reliability testing in military populations.[2] The diverse nature of the traumatic injuries present in SMs, as well as the occupational tasks the SMs are returning to, require more ecologically-valid assessments for determining function and making return to occupation decisions.[3,4]

The Assessment of Military Multitasking Performance (AMMP) has been one attempt at developing a military relevant assessment. The AMMP was designed to assess function and inform duty-readiness decisions in SMs who suffered traumatic brain injury.[5] While the AMMP has strong ecologic validity through the use of simulated military tasks, and has demonstrated clinically acceptable interrater reliability, it is only focused on the symptoms and deficits of a very specific TBI population. Thus, there is a need for a more generalized assessment that can be used to evaluate readiness and identify deficits across a broad range of domains and diagnoses.

Recognizing the need for a generalized assessment challenging both physical and cognitive abilities during military specific tasks, the Readiness Evaluation during simulated Dismounted Operations (REDOp) was developed at the Center for the Intrepid. The development leveraged over a decade of expertise caring for SMs and Veterans with traumatic extremity injuries and amputations at the Center for the Intrepid.[6–8]

The purpose of this study was to determine the intersession reliability of the REDOp and create a normative reference range of the embedded metrics for an able-bodied (AB) military population. Though the REDOp was developed to be generalizable to many patient populations, it was important to initially evaluate its effectiveness in one population. Lower extremity injuries are highly prevalent, have a large impact on readiness in the military[9], and can result in poor performance on physically demanding tasks.[10] Performance can further suffer on divided-attention and multi-tasks that combine physical demand with cognitive loads.[11,12] Thus, we further sought to determine the ability of the REDOp to identify deficits in SMs with a lower extremity injury (LEI).

## Methods

### Participants

Thirty-two AB individuals with no history of major musculoskeletal or neurologic injury, and twenty-two patients who had sustained a traumatic lower extremity injury recruited from a convenience sample of Service Members at Joint Base San Antonio participated in the REDOp (Table 1). Participants were required to be 18–55 years of age, eligible to receive care at a military hospital, able to ambulate unassisted for 20 continuous minutes, and had no other conditions that their safety or ability to complete the assessment. The LEI group all used their clinically prescribed orthopedic devices to participate in the REDOp (2 –prosthetic device, 19 –Intrepid Dynamic Exoskeletal Orthosis,[13,14] 1 –knee brace). Eighteen of the AB participants returned approximately 2 weeks (12.4±4.7 days) later to repeat the REDOp. This study was approved by the Regional Health Command-Central Institutional Review Board at Ft. Sam Houston, TX and all participants provided written informed consent prior to participation. The individual pictured in this manuscript has given written informed consent (as outlined in PLOS consent form) to publish the image.

**Table 1:** Participant characteristics. All values except Sex and Injury are given as Mean ± Standard Deviation (Range).

Characteristic	Able-Bodied	Lower Extremity Injury
Sex	24 M / 8 F	21 M / 1 F
Age (years)	27 ± 8 (18–51)	32 ± 7 (23–51)
Height (m)	1.76 ± 0.09 (1.56–1.94)	1.80 ± 0.09 (1.67–2.01)
Mass (kg)	76.0 ± 13.4 (50.9–95.3)	96.0 ± 18.2 (69.4–133.7)
Injury	NA	18 –Unilateral Limb Trauma
		2 –Bilateral Limb Trauma
		1 –Unilateral Transtibial Amputation
		1 –Unilateral Transfemoral Amputation

### REDOp

**Fig 1:**
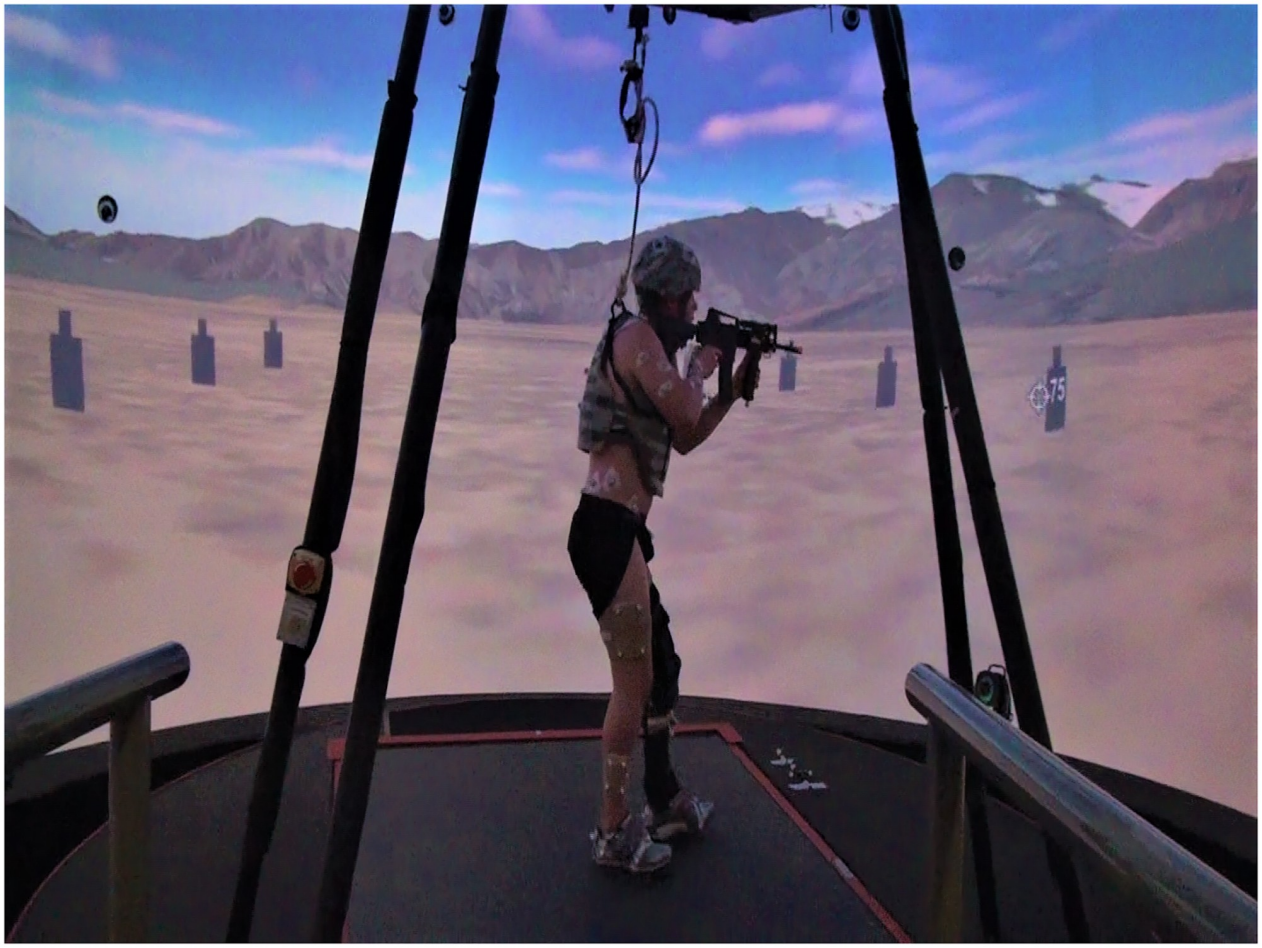
A participant with a left lower extremity injury engaging targets as part of the Readiness Evaluation during Simulated Dismounted Operations (REDOp) assessment.

Participants wore a Kevlar vest and helmet, and used a mock M4 rifle (total load ~11kg) during a simulated combat patrol performed on a treadmill embedded within a six degree-of-freedom motion platform in a virtual reality environment (Computer Assisted Rehabilitation ENviornment, Motekforce Link, Amsterdam, Netherlands). The REDOp consisted of walking over variable terrain (e.g. slopes and cross-slopes) on a flat-surface treadmill as speed and incline progressively increased (1.4 to 2.0 m/s and 3 to 10 degrees, respectively) over approximately 55 minutes, for a total distance of 4.5 km (Fig 1; S1 Video).

The REDOp is split into sequential blocks, where the participant walks over variable terrain, ascend a constant grade “hill”, and then encounter an ambush. The incline and speed of the “hill” portion increased each block from 1.4 to 2.0 m/s in 0.04 m/s increments and 3 to 10 degrees in 1 degree increments until blocks 9–12 where the speed remained at 2.0 m/s for all sections except the ambush. The ambush consisted of 20 E-type silhouette targets; 10 enemies (odd numbers) and 10 friendlies (even numbers). Participants had to acquire all targets and make shoot/don’t shoot determinations for each (shooting only the enemy targets) while walking at 0.6 m/s through the simulated ambush. At the end of each block, a 0–10 numerical pain scale was displayed to aid the participant in reporting their current pain magnitude and location. Participants progressed through the assessment until they requested to stop, were stopped by a member of the study team for safety concerns (e.g. reached age-predicted heart rate maximum (220-age),[15] pain increase of 4/10 or value greater than 5/10), or they completed the assessment. Prior to beginning the assessment, participants were oriented to the weapon and task, completed a practice standing ambush to become familiarized with the shooting component, then a walking trial to experience the variable terrain and walking through the ambush while engaging targets. Participants had the option to repeat any familiarization until they felt comfortable. Participant also completed an additional standing and walking ambush prior to starting the first block of the assessment.

### Data collection

During the assessment, kinematic data were collected at 120 Hz using a 30-camera motion capture system (Vicon Motion Systems, Oxford, UK). These cameras recorded the trajectories of 62 retro-reflective markers placed on the participants’ body segments, vest, helmet and the M4 rifle. A digitizing wand (C-Motion, Inc., Germantown, MD) was used to identify anatomical reference points used for joint center calculations.[16] Heart rate was monitored throughout the assessment using a chest-worn heart rate monitor (Polar Electro, Inc., Bethpage, NY).

### Data analysis

Marker trajectory data were initially digitized in Nexus (Vicon Motion Systems, Oxford, UK) and exported to Visual3D (C-Motion, Inc., Germantown, MD) for further analysis. Kinematic data were interpolated using a cubic spline and filtered at 6 Hz, with fourth order Butterworth low-pass filters. The marker data were used to create a 13-segment full body model, plus additional segments for the vest, helmet, and rifle. The model segments were scaled to properly account for participant height and mass, any orthoses or prostheses, and equipment sizes.

Total distance traveled was used as the measure of activity tolerance. Upon completion of the REDOp, the primary reason for stopping was classified as 1) endurance, 2) pain, 3) both (endurance and pain), or 4) completed patrol. To measure gait stability, whole-body angular momentum (H) about the model’s center of mass was calculated in the sagittal, frontal, and transverse planes.[17,18] Range of H was calculated as the difference between the maximum and minimum value across the entire gait cycle and normalized to height x mass x walking speed to make the value unitless and easier to compare between participants. This value was calculated for each step, then averaged across all steps as participants walked over variable terrain. A greater range of H indicates greater instability.[19]

Four measures of shooting performance were calculated using a custom MATLAB (MathWorks Inc., Natick, MA) script. Accuracy was calculated as the percent of correct responses (e.g. odd targets shot and even targets not shot) out of all responses. Precision was calculated as the percent of targets that were shot that were supposed to be shot (number of odd targets shot out of all odd targets). Reaction Time was calculated as the average time from when the target number was revealed to when it was shot. Acquisition Time was calculated as the total time from when the targets appeared to when the last target was identified.

### Statistical analysis

All statistics were performed using SPSS Statistics 22 (IBM Corporation, Armonk, NY, U.S.A.). The unadjusted criterion for statistical significance was set at p<0.05. Range of H and the shooting performance measures were only compiled for the first block; approximately 4 mins and 300 m of the assessment. Intersession reliability for range of H, distance, and reason for stopping were calculated using the subset of 18 AB participants that returned for a second session. Due to loss of data, only 17 AB participants’ data were used to calculate the intersession reliability of the shooting performance measures.

Intersession reliability for ordinal data was determined by calculating the interclass correlation coefficient (ICC) using a two-way random model (2, k) for consistency.[20] Intersession reliability for categorical data was determined using a Cohen’s Kappa calculation.[21] MDC values were also calculated for each measure.[22]

The Shapiro-Wilk test was used to evaluate data normality.[23] All measures for the AB group during both sessions were normally distributed with the exception of shooting accuracy and precision. Intersession differences in the AB group were evaluated using paired t-tests with a Bonferroni-Holm correction[24] for multiple comparisons and for categorical data using the McNemar-Bowker values.[25]

Data from the initial visit for the LEI participants were non-normally distributed, therefore Mann-Whitney *U* tests[26] were used to identify differences between the AB and LEI groups for quantitative data, and chi-square tests[27] were used for categorical data. Normative reference values were calculated for each variable as the minimum, maximum, and 5^th^, 25^th^, 50^th^, 75^th^, and 95^th^ percentiles for the AB participants.

## Results

**Fig 2:**
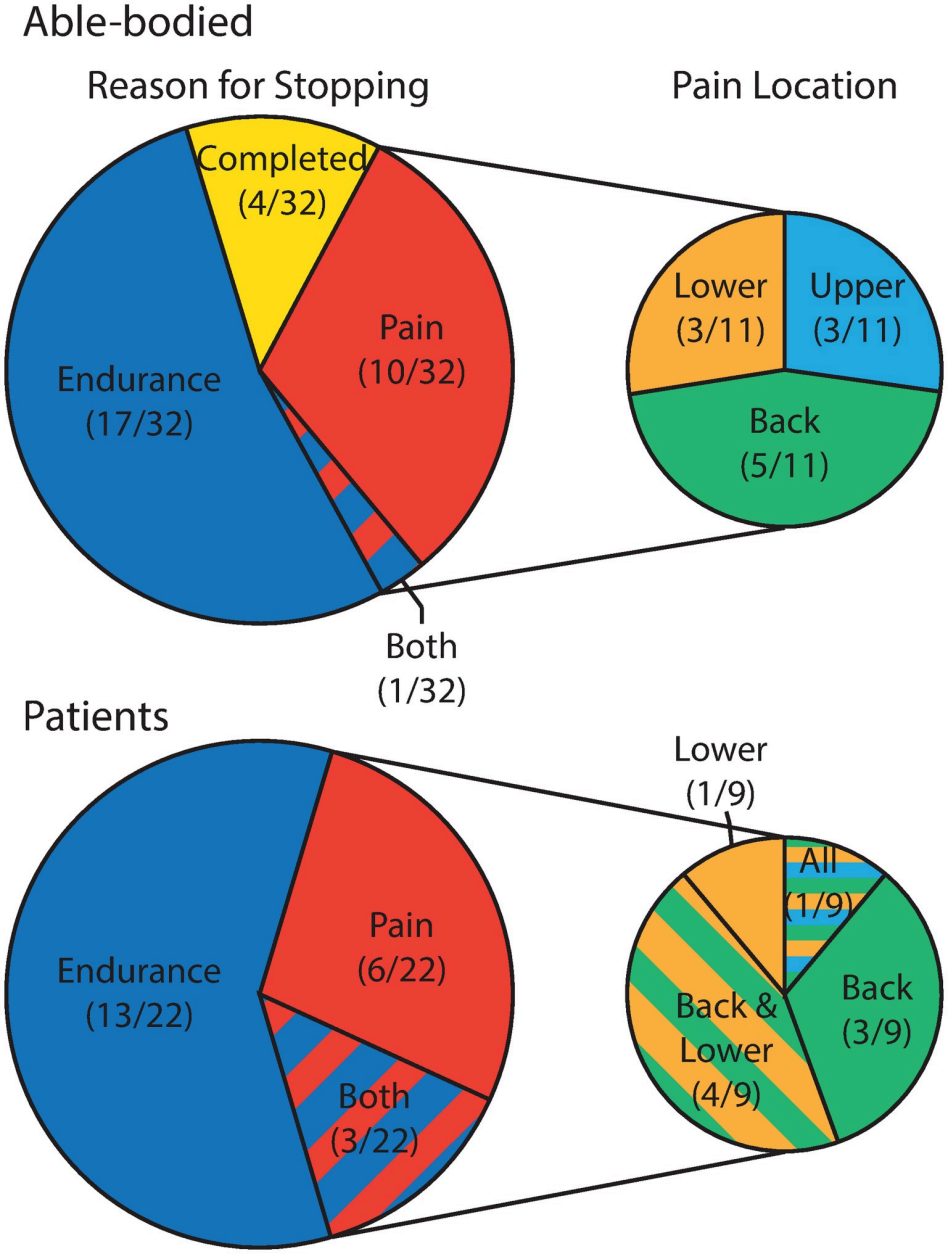
Pie charts showing the distributions of the primary reason for stopping and location of pain for the able-bodied and patient participants. The pain locations are only reported for the participants that stopped because of pain. The counts represent the presence of pain in any of those locations with some participants reporting pain in multiple areas.

Overall, the embedded performance variables were consistent between assessments showing strong intersession reliability in the AB group. The distance completed, our measure of activity tolerance, was not significantly different between sessions (Session 1: 2.98±1.03 km, Session 2: 3.37±1.20 km, p = 0.060) and demonstrated excellent intersession reliability (ICC = 0.91, MDC = 0.84 km). In Session 1, the primary performance limiter was cardiovascular endurance with 50% (9/18) of participants identifying it as the primary reason for stopping. Pain was the next most common limiter at 28% (5/18), and 11% (2/18) stopped due to some combination of both cardiovascular endurance and pain (Fig 2). The reasons for stopping had fair intersession reliability (kappa = 0.26) with no significant difference between sessions (p = 0.221).

The range of H, our measure of gait stability, showed excellent intersession reliability for all 3 planes (Sagittal: ICC = 0.88, MDC = 0.004; Frontal: ICC = 0.78, MDC = 0.010; Transverse: ICC = 0.93, MDC = 0.002). There was a slight increase in sagittal plane range of H during Session 2 (Session 1: 0.035±0.004, Session 2: 0.038±0.004, p = 0.001), but the difference was below the calculated MDC.

**Fig 3:**
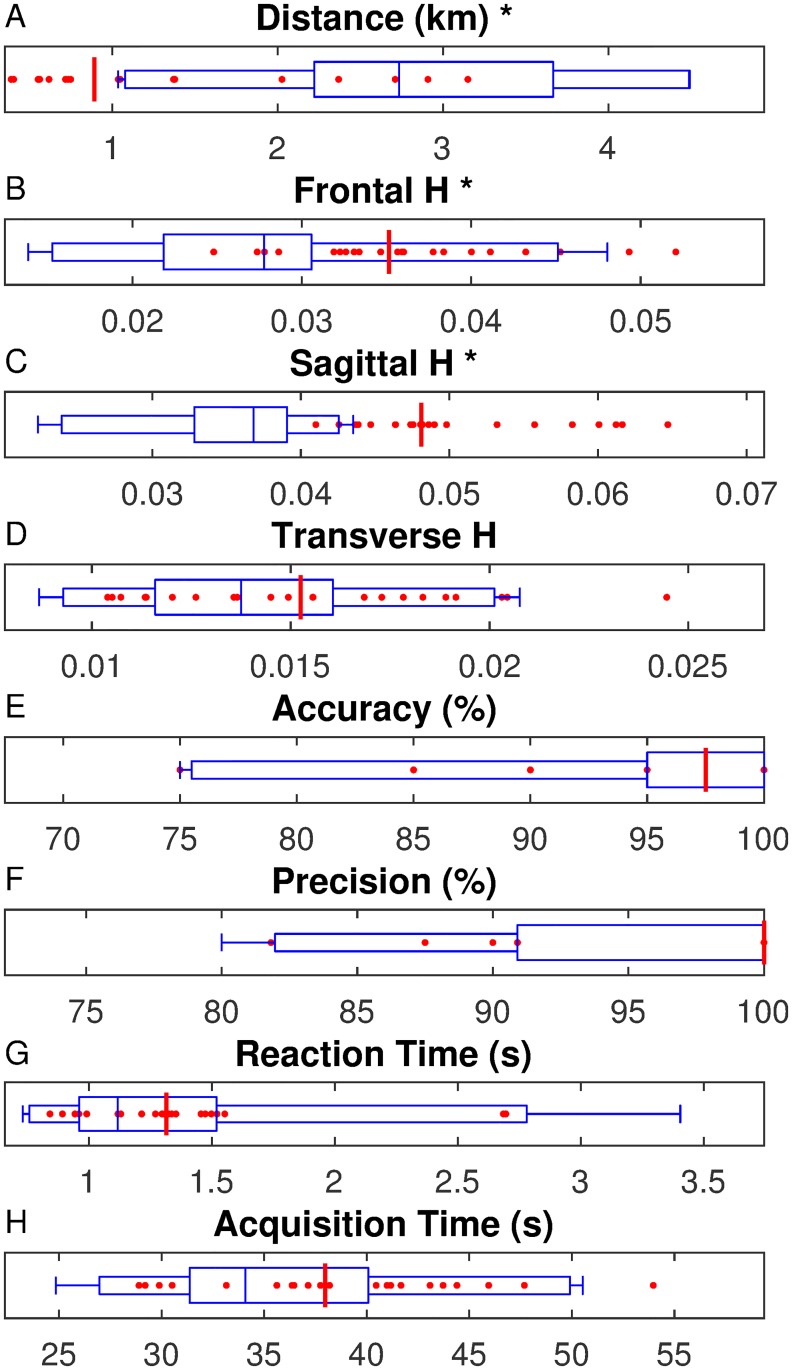
Patient data relative the normative reference range for the 8 performance metrics. The normative values are presented as a blue box plot with whiskers marking the maximum and minimum, small box indicating the 5^th^ and 95^th^ percentiles, and large box containing the median and first and third quartile as box ends. The median patient value is indicated by the red line with individual patient values plotted as red dots. * Indicates a significant difference between groups for the associated measure.

In general, the shooting variables were not as consistent as the other performance measures. Both Accuracy and Precision were significantly greater (p = 0.024) in Session 2 (99.1% and 98.9%, respectively) compared to Session 1 (95.3% and 95.8%, respectively), but the variables still had fair to good intersession reliability with differences below MDC values (Accuracy: ICC = 0.61, MDC = 9.6%; Precision: ICC = 0.47, MDC = 10.8%). While Reaction Time was not significantly different between sessions (p = 0.361), it had poor intersession reliability (ICC = 0.21, MDC = 0.72 s). Acquisition Time, on the other hand, was not significantly different between sessions and had excellent intersession reliability (ICC = 0.77, MDC = 9.82 s). Many of the variables were not normally distributed. As a result, the normative reference values were presented as box plots (Fig 3) and percentiles (Table 2) to show the distribution and for comparisons to patient data.

**Table 2:** Normative reference values of embedded metrics to include ICC and MDC values.

Metrics	Min	5%	25%	Median	75%	95%	Max	ICC	MDC
**Distance (km)**	1.04	1.08	2.22	2.73	3.67	4.5	4.5	0.91	0.84
**Frontal H**	0.014	0.015	0.022	0.028	0.031	0.045	0.048	0.78	0.010
**Sagittal H**	0.022	0.024	0.033	0.037	0.039	0.043	0.044	0.88	0.004
**Transverse H**	0.009	0.009	0.012	0.014	0.016	0.020	0.021	0.93	0.002
**Accuracy (%)**	75.0	75.5	95.0	95.0	100.0	100.0	100.0	0.61	9.6
**Precision (%)**	80.0	82.0	90.9	100.0	100.0	100.0	100.0	0.47	10.8
**Reaction Time (s)**	0.73	0.76	0.96	1.12	1.52	2.78	3.40	0.21	0.72
**Acquisition Time (s)**	24.84	26.98	31.37	34.08	40.07	49.91	50.53	0.77	9.82

The LEI group completed a significantly shorter distance (p<0.001) with a median distance of 0.89 km compared to 2.73 km for the AB group. Of the 22 LEI participants, 64% (14/22) were less than the 5^th^ percentile of the normative reference range for distance completed (Fig 3A). There was no difference in the reason for stopping between groups (p = 0.183). Only 12.5% (4/32) able-bodied participants completed all 4.5 km of the assessment (Fig 2) during Session 1. No one in the LEI group was able to complete the assessment (Fig 2). Endurance was the primary performance limiter for both groups reported by 53% (17/32) of the AB group and 59% (13/22) of the LEI group. Of the 11 AB participants and 9 LEI participants that reported pain as a reason for stopping, the most common location for the pain was in the lower back with 5/11 and 8/9, respectively (Fig 2).

The median for frontal plane range of H was 0.007 greater in the LEI group than the AB group (p<0.001), though the difference was less than the 0.01 MDC (Table 2, Fig 3B). Correspondingly, only 14% (3/22) of the LEI group had a range of H greater than the 95^th^ percentile. The range of sagittal plane H was also greater (0.048, p<0.001) in the LEI group than the AB group (0.037), with the difference greater than the MDC and 95% (21/22) of the LEI group above the 95^th^ percentile (Fig 3C). The range of transverse plane H and all of the shooting variables were not significantly different between groups (Fig 3D–3H).

## Discussion

Clinicians in military treatment facilities face the challenge of determining if maximal rehabilitation has been reached to allow discharge and a potential return to a SM’s occupation, but traditional physical performance measures and assessments do not simulate the demands associated with common warrior tasks. In practice, the REDOp will be performed by patients as an assessment of their function, with an individual’s performance compared to the normative reference range. The REDOp’s embedded measures of distance completed, angular momentum, and target acquisition time all demonstrated excellent intersession reliability. This suggests that the REDOp task and embedded measures were consistent at quantifying performance across all three domains: activity tolerance, gait stability, and shooting performance. In addition, the REDOp was able to identify deficits in activity tolerance and gait stability in individuals with LEIs compared to the AB group; which could be identified on a per-individual basis through comparison to the normative reference range.

### Activity tolerance

The REDOp incudes a physically demanding simulated dismounted patrol over hilly terrain. The intersession reliability was excellent for the distance completed. Only 4/32 AB participants completed the task during the first session, indicating that the task was sufficiently difficult to avoid ceiling effects. In addition to being difficult enough to challenge the highly fit AB SMs, the starting level and progression was implemented in such a way that even highly impaired individuals were able to complete 1 block, and it produced an adequate range of performance to create a useful normative reference range.

The reason(s) a person stopped the assessment provides clinical insight into the impairments that may prevent an individual’s return to occupational duties. The primary performance limiter for both groups was cardiovascular endurance, followed by musculoskeletal pain (Fig 2). A reduced level of activity can be expected following LEI; and together with the primary reason for stopping, may indicate deconditioning in this population. Lower extremity pain is also expected in the LEI population, as pain is common following lower extremity injury, and a primary reason for rehabilitative care and orthotic intervention.[28] Identifying the primary performance limiter for each individual could allow the treating clinician to further focus treatment, with the end goal of returning the individual to their pre-injury level of performance.

### Stability

The excellent intersession reliability of the embedded dynamic stability measures means that the REDOp can be effectively used to assess stability during destabilizing conditions as might be encountered during a military patrol. Dynamic instability has long been associated with lower extremity injuries during gait, especially under conditions which include perturbations.[18,29] SMs on dismounted patrols often operate in environments which are destabilizing[30] (e.g. variable terrain) and may suffer destabilizing perturbations associated with carrying loads[31,32] (e.g. weapon, vest, and helmet). These factors present a notable challenge to AB SMs, let alone injured SMs looking to return to their pre-injury occupation. Exposure to destabilizing environments or tasks during an assessment could help identify risk of falls which are reported to be the second leading cause of non-battle injuries air-evacuated from Operations Iraqi Freedom and Enduring Freedom.[33]

The LEI group was most unstable in the sagittal plane as all but 1 of the LEI participants had a range of H that was greater than the 95^th^ percentile. This is likely related to the reduced braking and propulsive abilities of the injured leg.[19] The use of prosthetic or orthotic devices and resulting reduced ankle control would further amplify dynamic instability when faced with destabilizing environmental challenges.

### Shooting

The ambushes incorporated into the REDOp evaluate shooting performance and decision making, where deficits during deployment could have dangerous consequences.[34] The task requires both physical and cognitive demands with simultaneous walking and targeting and the need to rapidly make shoot/don’t shoot determinations. This required decoupling of the upper and lower body to maintain a stable weapon regardless of the stability of the lower body. It was anticipated that the gait compensations and instability of the LEI participants would provide a greater challenge for effective shooting performance. However, neither the AB nor LEI group had a notable history of cognitive or upper extremity impairment; thus, the observed high accuracy and precision in both groups was not completely surprising. It is possible that the level of challenge was not sufficient to distinguish these populations, but differences would likely be detectible in patients with greater physical or cognitive injuries. While the AB participants appeared to improve in shooting accuracy and precision between sessions, the differences were below the calculated MDCs, and unlikely to impact the utility of the assessment. The small learning effect supports the importance of allowing participants to thoroughly familiarize themselves with the task prior to the first assessment.

In addition to providing reliable measures of shooting performance, participants anecdotally expressed satisfaction and enjoyment with performing this military-specific task. This enhanced participant engagement and the regular presentation of ambushes ensured continued attention throughout the assessment.

### Study limitations

While the REDOp has demonstrated strong military relevance, ecological validity and excellent reliability, there are barriers to widespread use. The primary impediment is limited access to virtual reality systems like the Computer Assisted Rehabilitation ENvironment. However, the Advanced Rehabilitation Centers in the U.S. Department of Defense, the primary locations where severely injured SMs receive care, and several foreign militaries, have access to similar systems.

The mass of the final participant samples was significantly different with the LEI group ~20 kg heavier than the AB group. There is the potential that this difference in mass between the groups contributed to the results. However, by normalizing the angular momentum measures to body mass, we minimized the effect that the difference in mass between the groups would have on the results. The significant differences in mass-normalized stability measures suggests that the assessment is able to identify functional differences between groups beyond just mass differences.

We evaluated the reliability of the REDOp in this study but did not formally evaluate validity. However, participants reported excellent ecologic and face validity of the REDOp, and it is aligned with Army doctrine. Ideally, during an engagement, SMs would take cover and fire from a stable firing position such as kneeling or prone. Due to constrains of the system, we were unable to simulate taking cover and dropping to the ground. However, the situation is still relevant as there are certain situations (e.g. close ambush) and units (some Special Forces groups) where the guidance is to continue moving and “fight through” the ambush. In our experience, assessment within the Computer Assisted Rehabilitation ENvironment has empowered patients who have withdrawn from their military identity during the rehabilitation process, and many find that they can successfully handle complex and challenging situations similar to those they experienced when deployed. However, formal validity testing is still necessary to determine how performance on the REDOp aligns with successful real-world performance.

## Conclusions

Overall, the REDOp was able to address the shortcomings of current assessments by providing a highly reliable, military-relevant assessment that can be used to measure performance and identify deficits across the domains of activity tolerance, gait stability, and shooting performance. This means that the REDOp assessment is both relevant to wounded SMs and their leadership in the tasks they are performing, and to the care team in the reliable, clinically relevant embedded metrics it provides. The excellent psychometric characteristics of the REDOp in healthy SMs support future work to evaluate the utility of REDOp for the evaluation of physical and cognitive function in a wider range of injured SMs. Additionally, continued collection of uninjured service members will allow for a more robust normative reference range and enable more granular comparison to include within specific occupational specialties. We expect REDOp to prove useful for evaluation and outcomes tracking, treatment planning, and readiness evaluation.

## Supporting information

S1 Video REDOp assessment. A participant with a left lower extremity injury negotiated variable terrain and engaging targets as part of the Readiness Evaluation during Simulated Dismounted Operations (REDOp) assessment.
(MP4)
